# Giant cell tumor of bone in pregnancy: A case series from the Eastern Asian Musculoskeletal Oncology Group

**DOI:** 10.1097/MD.0000000000048630

**Published:** 2026-05-15

**Authors:** Min Wook Joo, Jewoo Lee, Minpyo Lee, Yong-Suk Lee, Edward H.M. Wang, Akihiko Takeuchi, Chindanai Hongsaprabhas, Zhiping Deng, Seonhwa Jeong, Yang-Guk Chung

**Affiliations:** a Department of Orthopaedic Surgery, St. Vincent’s Hospital, College of Medicine, The Catholic University of Korea, Seoul, Korea; b Department of Orthopaedic Surgery, Incheon St. Mary’s Hospital, College of Medicine, The Catholic University of Korea, Seoul, Korea; c Department of Orthopedics, College of Medicine and Philippine General Hospital, University of the Philippines Manila, Manila, Philippines; d Department of Orthopaedic Surgery, School of Medicine, Kanazawa University, Kanazawa, Japan; e Department of Orthopaedic Surgery, Faculty of Medicine, Chulalongkorn University, Bangkok, Thailand; f Department of Orthopedic Oncology Surgery, Beijing Ji Shui Tan Hospital, Beijing, China; g Second Spring Women’s Clinic, Seoul, Korea; h Department of Orthopaedic Surgery, Seoul St. Mary’s Hospital, College of Medicine, The Catholic University of Korea, Seoul, Korea.

**Keywords:** case series, giant cell tumor, giant cell tumor of bone, pregnancy

## Abstract

Giant cell tumor of bone (GCTB) typically affects females of reproductive age and may be discovered during pregnancy. Pregnancy entails adaptations that could foster a microenvironment favorable to tumor growth. Although a few case reports have described aggressive features, larger studies in this setting are lacking. Thus, this study aimed to examine the clinical characteristics, obstetrical context, diagnostic process, therapeutic interventions, and prognostic outcomes of patients with GCTBs who presented during pregnancy. Patients were included if they developed pain or swelling during pregnancy at the site subsequently confirmed as primary or recurrent GCTB by pathology. Information on 8 patients was collected. The median age at presentation was 26.5 years (range, 22 to 37 years). One (1/8) patient experienced preterm delivery, and another (1/8) underwent elective termination of pregnancy before GCTB surgery. The first symptom was reported at a median gestational age of 5 + 6 (range, 2 + 3–20 + 2) by obstetric dating, but the first radiologic evaluations were often delayed. The most common location was the distal femur (3/8). Pathologic fracture occurred in one (1/8) patient, and all lesions showed Campanacci grade 3 features. The median lesion volume was 93.6 cm^3^ (range, 4.2–3348.6 cm^3^; interquartile range, 18.8–789.2 cm^3^). No pulmonary metastases were initially observed. Therapeutic approaches appeared to be tailored to individual clinical circumstances. No local recurrences developed in any patient with GCTB in the extremities. No malignant transformation occurred in any patient. Except for one (1/8) patient who died of another disease at 2.5-year follow-up, the other 7 patients (7/8) survived, and 6 (6/8) have remained continuously disease-free during a median follow-up of 5.3 years (range, 2.6–9.8 years). Clinicians should be aware of diagnostic imaging and treatment considerations to ensure timely and appropriate care for patients with GCTB during pregnancy. When musculoskeletal symptoms lack an obstetric explanation, prompt imaging might be crucial. For patients with tumors in surgically challenging sites, surveillance before pregnancy may be prudent. Further research is warranted to elucidate mechanisms through which pregnancy-related physiological changes may influence the oncologic behavior of GCTB.

## 1. Introduction

Giant cell tumor of bone (GCTB) is an intermediate lesion with International Classification of Diseases for Oncology/1 biological potential, representing roughly 5% of all primary bone tumors and 20% of nonmalignant bone tumors. It has an estimated incidence rate of <2 per 1,000,000 person-years. The tumor typically affects the end of long tubular bones in the mature skeleton, and in axial skeleton, it most commonly arises in the sacrum and vertebral bodies.^[1,2]^ It shows a slight female predominance. As most GCTBs occur between the ages of 20 and 45 years, which is generally considered to be the reproductive period, with around 10% of patients developing in their second decade, some lesions may be discovered incidentally during pregnancy.^[2]^

Pregnancy involves distinctive endocrinologic, immunologic, and angiogenic adaptations that could potentially create a microenvironment favorable to tumor growth in GCTBs.^[3]^ The literature commonly describes accelerated progression, aggressive recurrence, or markedly extensive tumors as characteristic features of these uncommon clinical conditions,^[3]^ along with accounts of individual diagnostic and therapeutic experiences.^[4-10]^ On the other hand, it has also been argued that these observations may merely reflect the fact that most patients are of reproductive age, rather than a true pregnancy-related effect.^[3]^

To the best of our knowledge, only a few case reports have documented GCTBs identified during pregnancy, likely because this situation is extremely rare.^[3]^ As case reports tend to emphasize atypical presentations, cases with more indolent features are likely underrepresented. To present diagnostic considerations and discuss therapeutic strategies, and to clarify relevant clinical characteristics that may help explore a potential association with pregnancy and ultimately investigate the underlying mechanisms that may pave the way for the development of pharmaceutical agents applicable to GCTBs in general, more studies involving a larger number of patients are required at present, since even those with a low level of evidence may yield insights beyond isolated case reports.

This study aimed to examine the clinical characteristics and obstetrical details of patients with GCTBs who presented during pregnancy, as well as the diagnostic process, therapeutic interventions, and prognostic outcomes.

## 2. Materials and methods

### 2.1. Study design

This study was a multi-national, multi-institutional, retrospective investigation, approved by the Institutional Review Board of St. Vincent’s Hospital, The Catholic University of Korea (VC21REGI0201). Participating institutions were recruited through the Eastern Asian Musculoskeletal Oncology Group. The study proposal was first introduced at in-person Eastern Asian Musculoskeletal Oncology Group meetings. Subsequently, detailed study information was circulated via email to all the members. A standardized preformed datasheet was used to ensure uniform data acquisition from the medical records of patients treated at 6 tertiary musculoskeletal tumor centers in 5 countries affiliated with The Catholic University of Korea (Korea), University of the Philippines Manila (Philippines), Kanazawa University (Japan), Chulalongkorn University (Thailand), and Beijing Ju Shui Tan Hospital (China). We reviewed the data on patients with GCTB who presented during pregnancy from 2010 to 2023.

### 2.2. Participants

The inclusion criteria were patients who developed pain or swelling during pregnancy at the site where primary or recurrent GCTB was subsequently confirmed by radiologic and pathologic evaluation during pregnancy or in the postgestational period (the first 42 days following delivery). All consecutive patients who met the inclusion criteria during the study period were included. The diagnosis was established according to the WHO Classification^[2]^ based on compatible imaging findings of a circumscribed osteolytic epiphyseal tumor in a skeletally mature individual, together with histologic confirmation showing numerous osteoclast-like giant cells admixed with a mononuclear stromal cell component without cytologic atypia. Patients with insufficient information were excluded during data acquisition. Finally, information on 8 patients was collected.

### 2.3. Variables

Data regarding age, past history of GCTB, pregnancy prognosis, delivery mode, gestational age (GA) at the first symptom such as pain or a palpable mass at lesion location, GA at the first radiologic evaluation, lesion location, pathologic fracture, Campanacci grade, lesion size and volume on computed tomography (CT) or magnetic resonance imaging (MRI), pulmonary metastasis at presentation on CT, treatment, gross residual lesion, local recurrence, malignant transformation,^[11]^ oncologic result, and the follow-up period were collected. Age was defined as the patient’s age at symptom onset during pregnancy. The Campanacci grade^[12]^ is a radiographic classification of GCTB based on lesion margins, cortical integrity, and soft-tissue extension, reflecting the biological aggressiveness of the tumor: grade I lesions are characterized by well-defined margins with an intact cortex; grade II lesions show relatively well-defined margins with cortical thinning or expansion; and grade III lesions demonstrate ill-defined margins with cortical destruction and soft-tissue extension. Tumor volume was estimated by ellipsoidal approximation based on 3 orthogonal tumor dimensions measured on CT or MRI.^[13]^ Patients who received non-surgical treatments or whose lesions were grossly deemed incompletely removed during surgery were classified as having gross residual lesions. Oncologic results were defined as patients’ disease status at the last follow-up and reported as continuously disease-free, alive with disease, or died of another disease.

### 2.4. Statistical analysis

Continuous variables are described using medians and ranges while categorical variables are presented as counts and proportions. Interquartile range was used for volume of tumor in addition. GAs are depicted in weeks + days, following standard obstetric convention.

## 3. Results

**Figure 1.**
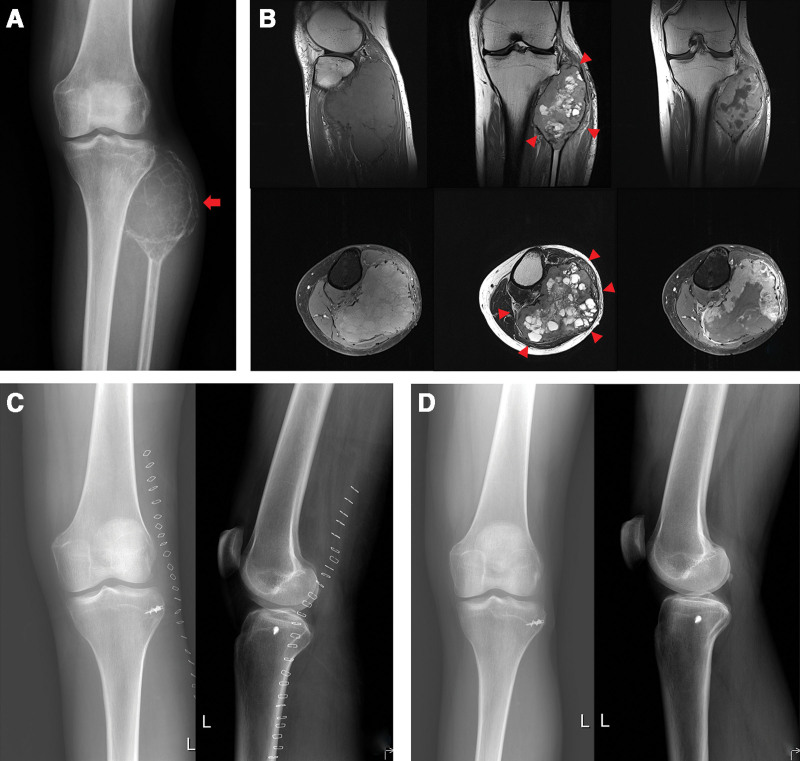
A female (patient 2 in Tables 1 and 2), who was 37 years old at diagnosis of giant cell tumor of bone (GCTB) in the left proximal fibula, reported no symptoms before pregnancy. However, after becoming pregnant, she noticed a rapid enlargement of a lump near her knee. (A) An anteroposterior plain radiograph of both knees showed an expansile bone lesion (an arrow). (B) Coronal and axial magnetic resonance images demonstrated GCTB with extraosseous extension (arrowheads). (C) Radical resection and re-attachment of the lateral collateral ligament to the proximal tibia were performed. (D) No local recurrence was detected 4 years after surgery. GCTB = giant cell tumor of bone.

Demographic, obstetrical, and GCTB-related details of the patients are presented in Table 1. The median patient age at presentation was 26.5 years (range, 22–37 years). One patient (1/8) requested preterm delivery due to intolerable back pain, with no apparent obstetric cause identified, and another (1/8) opted for artificial abortion before surgery for GCTB (Fig. 1). The first symptom was reported at a median GA of 5 + 6 (range, 2 + 3–20 + 2), but the first radiologic evaluations were often delayed despite persistent symptoms. The most common location was the distal femur (3/8). Among primary lesions, the distal femur was the most frequently affected site (3/6), whereas 2 recurrent ones involved the thoracic vertebra and proximal tibia. Pathologic fracture occurred in one (1/8) patient, and all lesions showed Campanacci grade 3 features. The median lesion volume among all patients was 93.6 cm^3^ (range, 4.2–3348.6 cm^3^; interquartile range, 18.8–789.2 cm^3^), including 1 patient with a pathologic fracture, for whom the volume was retrospectively estimated based on its presumed dimensions before the fracture occurred. No pulmonary metastases were observed on initial chest CT scan obtained after presentation.

**Table 1:** Patient demographic, obstetrical, and tumor-related details.

Patient number	Age (yr)	Past history of GCTB	Prognosis of pregnancy	Delivery mode	First symptom (GA)	First radiologic evaluation (GA)	Location	Pathologic fracture (GA)	Campanacci grade	Size (CT or MRI)	Initial pulmonary metastasis
1	27	+	P	C	Pain 13 + 0	28 + 3	Thoracic vertebra	–	3	15.9 × 8.8 × 12.6 cm (MRI)	–
2	37	−	A	N/A	Lump 5 + 0	5 + 4	Proximal fibula	–	3	11.3 × 8.4 × 7.8 cm (MRI)	–
3	25	−	F	C	Pain 2 + 3	3 + 0	Distal femur	–	3	2.3 × 3.9 × 4.5 cm (CT)	–
4	22	−	F	C	Lump 20 + 2	28 + 4	Distal radius	–	3	3.2 × 2.1 × 1.2 cm (MRI)	–
5	33	−	F	V	Pain 3 + 0	24 + 0	Distal femur	–	3	5.7 × 4.5 × 4.4 cm (MRI)	–
6	28	−	F	C	Pain 6 + 0	30 + 0	Distal femur	30 + 0	3	8.4 × 5.6 × 5.2 cm (CT)	–
7	26	+	F	V	Pain 4 + 0	Postpartum	Proximal tibia	–	3	4.4 × 2.8 × 2.8 cm (MRI)	–
8	25	−	F	V	Lump 16 + 0	Postpartum	Ilium	–	3	19 × 18 × 18.7 cm (CT)	–

Age is defined as the patient’s age at symptom onset during pregnancy.

The size of the lesion with a pathologic fracture was estimated based on the presumed dimensions prior to fracture.

A = artificial abortion, C = cesarean section, CT = computed tomography, F = full-term birth, GA = gestational age, GCTB = giant cell tumor of bone, P = preterm birth, MRI = magnetic resonance imaging, N/A = not applicable, V = vaginal delivery.

**Table 2:** Treatment and prognosis of giant cell tumor of bone.

Patient number	Treatment		Prognosis					Follow-up (yr)
	Surgery	Medicine	Gross residual lesion	Local recurrence	Malignant transformation	Pulmonary metastasis	Oncologic result	
1	R	BP	+	N/A	–	+	AWD	13.3
2	R	–	−	–	–	−	CDF	6
3	C	–	−	–	–	−	CDF	2.6
4	C	–	−	–	–	−	CDF	8.4
5	C	–	−	–	–	−	CDF	4.5
6	C	–	−	–	–	−	CDF	4
7	R	D	−	–	–	−	CDF	9.8
8	-	D	+	N/A	–	−	DOAD	2.5

Patients who received non-surgical treatments or whose lesions were grossly deemed incompletely removed during surgery were classified as having gross residual lesions.

AWD = alive with disease, BP = bisphosphonate, C = curettage, CDF = continuously disease-free, D = denosumab, DOAD = died of another disease, GCTB = giant cell tumor of bone, N/A = not applicable, R = resection.

**Figure 2.**
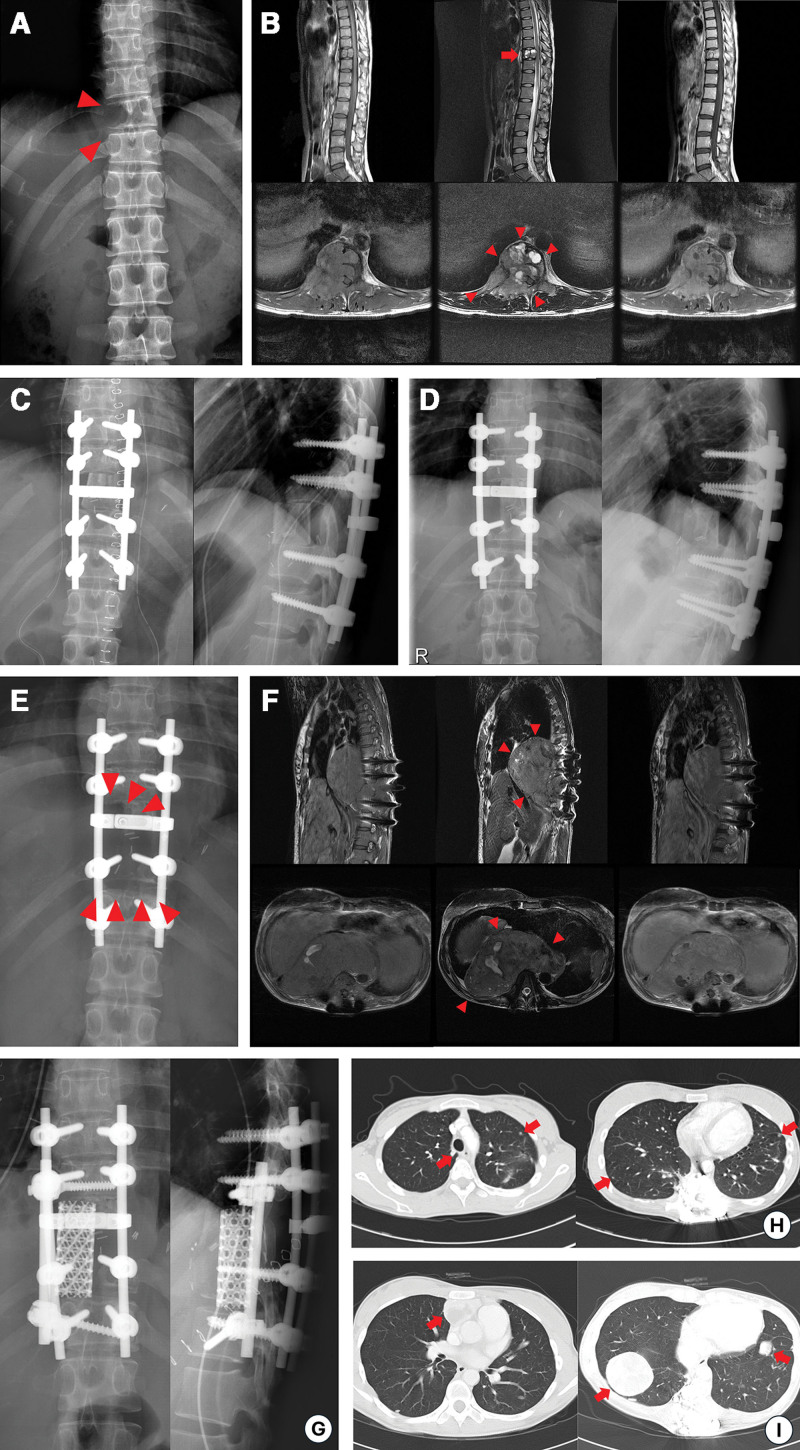
A female who was 25 years old at initial diagnosis of the primary giant cell tumor of bone (GCTB) in the 10th thoracic vertebra (patient 1 in Tables 1 and 2). (A) An anteroposterior (AP) plain radiograph shows bone resorption in the right side of the vertebral body (arrowheads). (B) Sagittal and axial magnetic resonance images demonstrated a Campanacci grade 3 GCTB (an arrow and arrowheads). One year and ten months after (C) resection, reconstruction with a structural allograft, and posterior fusion and instrumentation, (D) No local recurrence was observed. Thus, she decided to get pregnant. (E) At the gestational age of 28 + 3, at postoperative 2 years and 8 months, bone resorption (arrowheads) was revealed on AP and lateral radiographs. (F) A huge, recurrent GCTB (arrowheads) was detected on follow-up magnetic resonance images 2 years and 9 months after surgery. (G) The second resection, reconstruction with a metal cage, and posterior and lateral instrumentation were performed. (H) Multiple nodules in the bilateral lung fields (arrows) were detected on axial computed tomography (CT) images of the chest 5 months after the second surgery. (I) Follow-up CT images demonstrated markedly enlarged lesions (arrows), the 2 hugest ones of which were histologically confirmed to be pulmonary implants. AP = anteroposterior, CT = computed tomography, GCTB = giant cell tumor of bone.

Treatment and patients’ prognoses are summarized in Table 2. While the patient with a recurrent thoracic vertebra lesion was surgically treated after preterm delivery at roughly 28 weeks of gestation, gross residual disease remained after surgery, and pulmonary metastases subsequently developed (Fig. 2). Beginning 2 years post-operatively, she received intravenous zoledronate and ibandronate, and oral risedronate over the following 10 years. Osteonecrosis of the jaw was diagnosed 1 year after discontinuation. She remains alive with disease at last follow-up. As a representative example, based on the first symptom and imaging reported, the estimated average growth rate was ~4.5 cm^3^/d, although precise growth kinetics cannot be determined without interval imaging. Another one with the iliac lesion treated only with palliative denosumab administration, which was initiated postpartum since its use is contraindicated during pregnancy.^[14]^ In addition, 1 patient who underwent resection also received denosumab, which was likewise initiated postpartum. No local recurrences developed in any patient with GCTB in the extremities. No malignant transformation occurred in any patient. Except for one (1/8) patient who died of another disease at a follow-up of 2.5 years, the other 7 patients (7/8) survived, and 6 (6/8) have remained continuously disease-free during a median follow-up period of 5.3 years (range, 2.6–9.8 years).

## 4. Discussion

GCTB may affect females of childbearing age, and although rare, some lesions develop, progress, or recur during pregnancy.^[2]^ Physiologic adaptations during pregnancy could influence a microenvironment in ways that favor tumor growth.^[3]^ A limited number of case reports have described fulminant clinical features in such instances.^[3]^ At present, studies with even modestly larger patient numbers are needed to clarify these characteristics, explore potential links to pregnancy, and ultimately provide preliminary insights into the underlying mechanisms that may ultimately guide the development of therapeutic agents broadly applicable to GCTBs. In this multi-national, multi-institutional case series, we investigated the clinical profiles and obstetrical features of patients with GCTBs who presented during pregnancy, and explored their diagnostic pathways, treatment approaches, and clinical outcomes. The age distribution of patients was similar to that of the general GCTB population, with adolescents and the elderly notably unrepresented.^[2]^ Imaging evaluations were mostly postponed following symptom onset. The lesion distribution in pregnant patients was similar to that in non-pregnant patients with GCTB, showing a predilection for the distal femur.^[2]^ Some tumors were indeed markedly large, consistent with previous reports,^[3]^ while tumor sizes varied across lesions. No evidence of pulmonary metastases was initially detected. Both surgical and systemic therapeutic approaches appeared to be tailored to individual clinical circumstances. As no local recurrence was noted in patients with GCTB in the extremities, the oncologic outcomes were favorable, and no malignant transformation was observed.

This study had several limitations. Despite its multi-national and multi-institutional design, only limited data were collected from patients with heterogeneous characteristics. Due to the rarity of the condition, patients were included regardless of the timing of diagnosis and treatment. Over the study period, particularly during the era of advanced imaging technologies and denosumab,^[15,16]^ diagnostic methods and therapeutic strategies have evolved, and patients may have received markedly different approaches. Nevertheless, as with other rare musculoskeletal tumors, it is often difficult to draw meaningful conclusions from data collected at a single institution. Therefore, multi-center collaboration is essential. Second, significant differences in the socioeconomic and cultural contexts of the countries of the contributing institutions must be acknowledged. These factors may influence diagnostic and treatment decisions. Even so, regional cooperation remains crucial, especially for identifying potential ethnic differences.

Unique endocrinologic, immunologic, and angiogenic changes during pregnancy may foster a tumorigenic microenvironment in GCTBs.^[3]^ The roles of estrogen and progesterone receptors in GCTBs currently remain controversial.^[6,17-19]^ During pregnancy, progesterone also modulates immune tolerance by affecting regulatory immune cells, which may overlap with immune escape mechanisms seen in aggressive GCTBs.^[20]^ In addition, elevated beta subunit of human chorionic gonadotropin during pregnancy may act as an autocrine/paracrine growth factor in GCTBs, potentially interfering with apoptotic signaling pathways.^[3]^ The presence of fetal antigens and shared oncofetal antigens could contribute to tumor immune escape, while no direct evidence of oncofetal antigen expression in GCTBs has been observed.^[3]^ Macrophage-colony stimulating factor, crucial in both fetal-maternal immune modulation and osteoclast activation, may further link pregnancy-related biology to GCTB behavior.^[21]^ Angiogenic factors such as vascular endothelial growth factor and placental growth hormone, essential for placental vascular development, are expressed in GCTBs as well and may promote osteoclastogenesis and tumor progression.^[22]^ Alternatively, it has been claimed that such features of GCTBs during pregnancy may be only a coincidence since affected patients are simply of childbearing age.^[3]^ While several lesions in this study were extremely extensive, consistent with previous reports on GCTB during pregnancy,^[3]^ a few others were not markedly enlarged despite prolonged symptom durations. This discrepancy may reflect the complex interplay of pregnancy-associated physiologic changes, which likely influence tumor behavior to varying degrees. Since case reports typically highlight atypical manifestations, those with more indolent or unremarkable features probably remain underrepresented in the literature.

Diagnostic delays might have resulted from the misinterpretation of GCTB symptoms, particularly spinal lesions, as being related to pregnancy.^[9]^ In addition, as a lack of knowledge about the health consequences of radiation exposure may lead to unfounded concern, clinicians who care for pregnant patients often overestimate the teratogenic risk associated with diagnostic imaging. The clinical consensus is that a single diagnostic imaging procedure does not increase the risk of fetal anomalies or pregnancy loss.^[23]^ The potential effects of radiation primarily depend on the dose and gestational stage at the time of exposure. The fetus is most vulnerable to ionizing radiation during organogenesis (~2–7 weeks post-conception) and in the early fetal period (8–15 weeks post-conception). However, noncancer health effects have not been observed at any gestational stage following fetal exposure to ionizing radiation doses below 0.05 Gy (5 rad). Most x-ray examinations involve radiation doses of <50 mSv, with no significant effect on fetal growth and development. Radiation risks are deemed to be low for doses up to 100 mSv compared with the normal risks of pregnancy.^[23]^ While abdominal shielding was traditionally used to protect the fetus, recent evidence suggests that most fetal radiation exposure results from internal scattering, rendering such shielding of limited value and, in some instances, it may paradoxically interfere with automatic exposure control, increasing the overall radiation dose.^[24]^ MRI, which uses nonionizing radiation, is considered safe during pregnancy, as long as the use of gadolinium-based contrast agents is generally avoided unless absolutely necessary, due to the uncertain effects on the fetus.^[25,26]^

In situations where surgical treatment under general anesthesia is mandatory for GCTB during pregnancy, pregnancy termination, as in Patient 2 in our study, should not be regarded as a mandatory prerequisite for surgery.^[27]^ The following generalizations may assist in decision making regarding surgery under general anesthesia during pregnancy: Current anesthetic agents, when administered at standard concentration, show no teratogenic effects at any GA. Furthermore, no evidence supports the claim that *in utero* exposure to anesthetic or sedative medications harms fetal brain development. It is also broadly recommended that medically required surgery should not be denied or postponed at any stage of pregnancy, as it may pose risks to both the patient and the fetus. The patient’s primary obstetric provider should be informed when planning surgical management for GCTB. If that provider is not available, an alternative obstetrician with privileges at that institution should be involved. If fetal monitoring is indicated, the surgery should be performed at an institution with neonatal and pediatric support, with immediate accessibility to an obstetrician capable of cesarean section and a qualified staff member for fetal monitoring.^[27]^

Two patients received denosumab only in the postpartum period in our series. Denosumab, a monoclonal antibody against receptor activator of nuclear factor kappa-B ligand, has become an important therapeutic agent for GCTB.^[28]^ However, denosumab is not recommended during pregnancy; it may cause fetal harm based on its mechanism of action and animal study data. Experimental studies in knockout mice have shown fetal lymph node agenesis, impaired dentition, and abnormal bone growth, while altered maturation of maternal mammary glands led to impaired lactation postpartum. Although no maternal or fetal toxicity was observed in cynomolgus monkeys during early gestation, transplacental transfer of monoclonal antibodies increases during the second and third trimesters, raising concerns about potential adverse fetal effects. Therefore, denosumab should be avoided during pregnancy, and its initiation deferred until after delivery.^[14]^

## 5. Conclusion

In this study, we found that the age range and lesion locations of patients with GCTB who presented during pregnancy were broadly similar to those of the general population with GCTB. Imaging evaluations were often delayed despite the early onset of symptoms. Some tumors were massively enlarged, consistent with certain previous reports, although this was not the case for all patients. No pulmonary metastases were observed at the initial presentation. Treatment strategies were individualized, and the oncologic outcomes were favorable overall, with no malignant transformation observed and no local recurrence in those with GCTB in the extremities. It may be helpful for clinicians to provide accurate information regarding diagnostic imaging, particularly when musculoskeletal symptoms such as pain or lump arise without obstetric explanation, and to offer guidance on treatment options once a diagnosis is made, in order to support timely and appropriate care. Adequate surveillance could be advisable before planning a pregnancy, especially when the tumor is situated where complete surgical removal is demanding. Further research may help elucidate potential mechanisms through which pregnancy-related changes could influence the oncologic behavior of GCTB during pregnancy.

## Author contributions

**Conceptualization:** Min Wook Joo, Yang-Guk Chung.

**Data curation:** Min Wook Joo, Yong-Suk Lee, Edward H.M. Wang, Akihiko Takeuchi, Chindanai Hongsaprabhas, Zhiping Deng.

**Formal analysis:** Min Wook Joo, Jewoo Lee, Minpyo Lee.

**Investigation:** Min Wook Joo, Yong-Suk Lee, Edward H.M. Wang, Akihiko Takeuchi, Chindanai Hongsaprabhas, Zhiping Deng.

**Methodology:** Min Wook Joo, Yang-Guk Chung.

**Project administration:** Min Wook Joo.

**Software:** Jewoo Lee, Minpyo Lee.

**Supervision:** Seonhwa Jeong.

**Validation:** Min Wook Joo, Yong-Suk Lee, Seonhwa Jeong, Yang-Guk Chung.

**Writing – original draft:** Min Wook Joo.

**Writing – review & editing:** Min Wook Joo, Yong-Suk Lee, Seonhwa Jeong.
